# Phase-locked closed-loop ultrasound stimulation modulates theta and gamma rhythms in the mouse hippocampus

**DOI:** 10.3389/fnins.2022.994570

**Published:** 2022-09-08

**Authors:** Zhenyu Xie, Jiaqing Yan, Shuxun Dong, Hui Ji, Yi Yuan

**Affiliations:** ^1^School of Electrical Engineering, Yanshan University, Qinhuangdao, China; ^2^Key Laboratory of Intelligent Rehabilitation and Neuromodulation of Hebei Province, Yanshan University, Qinhuangdao, China; ^3^College of Electrical and Control Engineering, North China University of Technology, Beijing, China; ^4^Department of Neurology, The Second Hospital of Hebei Medical University, Shijiazhuang, China

**Keywords:** closed-loop, ultrasound stimulation, theta rhythm, gamma rhythm, ultrasound pressure

## Abstract

Previous studies have demonstrated that open-loop transcranial ultrasound stimulation (TUS) can modulate theta and gamma rhythms of the local field potentials (LFPs) in the mouse hippocampus; however, the manner in which closed-loop TUS with different pressures based on phase-locking of theta rhythms modulates theta and gamma rhythm remains unclear. In this study, we established a closed-loop TUS system, which can perform closed-loop TUS by predicting the peaks and troughs of the theta rhythm. Comparison of the power, sample entropy and complexity, and phase-amplitude coupling (PAC) between the theta and gamma rhythms under peak and trough stimulation of the theta rhythm revealed the following: (1) the variation in the absolute power of the gamma rhythm and the relative power of the theta rhythm under TUS at 0.6–0.8 MPa differ between peak and trough stimulation; (2) the relationship of the sample entropy of the theta and gamma rhythms with ultrasound pressure depends on peak and trough stimulation; and (3) peak and trough stimulation affect the PAC strength between the theta and gamma rhythm as a function of ultrasound pressure. These results demonstrate that the modulation of the theta and gamma rhythms by ultrasound pressure depends on peak and trough stimulation of the theta rhythm in the mouse hippocampus.

## Introduction

Neural oscillatory activity refers to continuous and rhythmic neural activity of neurons in the brain, which plays an important role in the information processing of neural networks (Ward, 2003; Buzsaki and Draguhn, 2004; Uhlhaas and Singer, 2010). Previous studies have found that neural oscillatory activity in specific frequency bands, including theta (4–8 Hz), alpha (9–13 Hz), beta (14–30 Hz), and gamma (above 30 Hz), is associated with learning and memory performance. Theta oscillation (4–8 Hz) plays a key role in learning, spatial encoding, memory, and sniffing movement (Düzel et al., 2010; Fell and Axmacher, 2011; Hsieh et al., 2011). Some researchers have proposed that the theta rhythm is induced by the hippocampal cortical pathway, which subsequently enters the neurons in different cortical areas, and connects the separated neurons through synchronous oscillatory activity for information transmission processing. The establishment of this connection forms the physiological basis for working memory and encoding of new information (Colgin, 2013; Kropff et al., 2021; Nuñez and Buño, 2021). The gamma rhythm represents fast oscillatory activity of neurons and neuron groups, which is mainly generated by the network composed of inhibitory interneurons and can facilitate synaptic transmission and modulate sensory cognitive activities, such as attention and memory tasks (Colgin and Moser, 2010; Buzsáki and Wang, 2012; Mably and Colgin, 2018). Theta and gamma rhythms play an important role in the evaluation of external stimuli, such as optogenetic stimulation, deep brain stimulation, transcranial magnetic stimulation, etc. (Mangia et al., 2014; Noda et al., 2018; Etter et al., 2019).

Low-intensity transcranial ultrasound stimulation (TUS), which has recently emerged as a non-invasive neuromodulation technique, possesses high spatial resolution and the ability to access deep structures of the brain (Bystritsky et al., 2011; Niu et al., 2018; Yu et al., 2020). In the past decade, low-intensity TUS has been widely used in the field of neuromodulation (Jiang et al., 2018; Baek et al., 2020; Yuan et al., 2020). Previous studies have reported the ability of TUS to elicit the encoding of neural information in the cortical and deep brain regions, especially theta and gamma rhythms of local field potentials (LFPs). For example, studies have shown that the relative power in the theta (4–8 Hz) frequency band of the mouse motor cortex under open-loop ultrasound stimulation decreases with the increase in ultrasound pressure at 0–0.5 and 0.5–1 s, the relative power in the gamma (30–45 Hz) band increases with the increase in ultrasound pressure and stimulation duration (Wang et al., 2019).

Ultrasound stimulation of the hippocampus significantly enhances both signal pressure of the gamma band (Tufail et al., 2010) and power pressure in the gamma band in the stimulation area (Yu et al., 2016). Studies have also shown that TUS significantly modulates the phase-amplitude coupling (PAC) strength between the theta and gamma bands in the rat hippocampus, which increases with ultrasound pressure (Yuan et al., 2016a,b). We also found that TUS of the thalamus enhances the amplitude of the theta rhythm of the thalamus and the pressure of the theta rhythm in the motor cortex (Wang et al., 2021). Another study showed that open-loop ultrasound stimulation alters the phase distribution of intrinsic brain activity at the beta frequency, but not at gamma frequency. This modulation is accompanied by changes in the phase rate of the beta and gamma frequencies (Mueller et al., 2014). In conclusion, open-loop ultrasound can significantly modulate theta and gamma rhythms of LFPs in different brain regions including the cortex, hippocampus, and thalamus. Closed-loop ultrasound stimulation better enables perform phase-locked neuromodulation according to the characteristics of the signal compared to open-loop stimulation. In previous studies, we found that peak and trough stimulation of the theta rhythm can enhance the power of the theta rhythm (Yang et al., 2020). However, the manner in which TUS with peak and trough stimulation of the theta rhythm modulates the gamma rhythm and PAC between the theta and gamma rhythms remains unelucidated.

Ultrasound parameters play a key role in ultrasound stimulation. Previous research has proven that modifying the parameters of ultrasound radiation pressure (such as frequency, pressure, duty cycle, etc.) can elicit different ultrasound neuromodulation functions. In open-loop TUS, ultrasound pressure has a significant effect on the stimulation results with respect to the motor response, neural firing, cerebral hemodynamics, etc. For example, the pressure of ultrasound stimulation is correlated with the observed robustness of the motor response with the increase in ultrasound pressure, and the amplitude of the motor-responsive electromyogram signal decreases with the increase in ultrasound pressure (Tufail et al., 2010; Mehiæ et al., 2014). The strength of the calcium response and neural response evoked by ultrasound neuronal stimulation increases with the increase in ultrasound pressure (Qiu et al., 2019; Yoo et al., 2022). Moreover, the current pressure of ultrasound-induced TWIK-related arachidonic acid activated K^+^ (TRAAK) channels increases with the surge in ultrasound pressure (Sorum et al., 2021). The number of ultrasound-evoked spikes in I92L-infected neurons is dependent on the peak negative pressure associated with the increase in ultrasound stimulation pressure (Ye et al., 2018), and the coupling strength between neural oscillations and hemodynamics exhibits a linear increase with an increase in ultrasound pressure (Yuan et al., 2021). In conclusion, the modulation effect of open-loop ultrasound stimulation on neural activity depends on ultrasound pressure. However, until now, the manner in which neural firing activity, including the theta and gamma rhythms, varies with ultrasound pressure under phase-locked TUS remains unknown.

Therefore, we conducted this study to obtain answers to the above-mentioned questions, and to this end, established a closed-loop TUS system that can accurately track the peaks and troughs of the theta rhythm. We recoded the LFPs of the stimulation area under closed-loop TUS. Thereafter, we analyzed the power spectrum, complexity, sample entropy, and PAC strength of the theta and gamma rhythms under peak-to-trough stimulation as a function of ultrasound pressure.

## Materials and methods

### Animals and groups

Sixteen mice (C57BL/6, male, body weight: 20–25 g, Beijing Weitong Lihua Laboratory Animal Technology Co., Ltd., China) were used in this study. All procedures were conducted in accordance with the relevant regulations of animal ethics and the Ethics Committee of Yanshan University. The mice were housed in standard cages under a light/dark cycle of 12-h/12-h and provided food and water *ad libitum*. The mice were randomly divided into the peak stimulation (8 mice) and trough stimulation groups (8 mice).

### Operation

General anesthesia was induced with isoflurane 2% during the procedure. After administering anesthesia in the induction box, the mice were fixed on an adapter (68030, Reward Company, China) and placed on a stereotaxic device (68001, Reward Company, China). The anesthesia mask of the gas anesthesia machine (R540 mobile small animal gas anesthesia machine, Reward Company, China) was placed over the mouse’s mouth for real-time anesthesia. The fur covering the animal’s skull was shaved, and the skin was cleaned with physiological 0.9% sodium chloride solution. The scalp was incised along the midline of the skull, and the subcutaneous tissue and periosteum were removed in preparation for the experiment. A hole with diameter of 0.5 mm was drilled at the following coordinates relative to the bregma: anteroposterior (AP) = –2 mm, medial-lateral (ML) = 2 mm, and dorsoventral (DV) = –1.5 mm. A tungsten microelectrode (WE50030.1B10, MicroProbe, United States) was used to record the LFP signals. Two holes were drilled in the nasal bone to fix the ground and reference electrodes. During the experiment, all mice were anesthetized using 0.3% isoflurane.

### Closed-loop ultrasound stimulation system and ultrasound parameters

**FIGURE 1:**
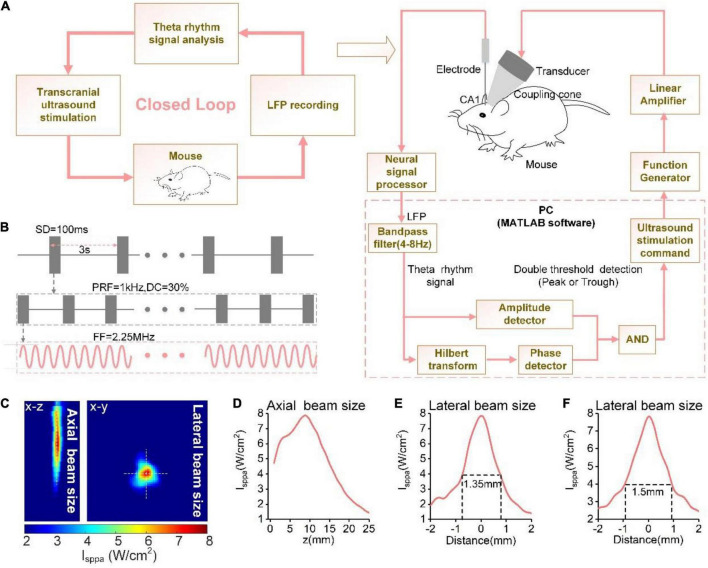
**(A)** The schematic of the phase-locked close-loop ultrasound stimulation system. **(B)** Time sequence of ultrasound stimulation. **(C)** Ultrasound field distribution under the skull in xy plane. The reconstruction profile along the blue dotted lines. Ultrasound field distribution under the skull in the xz and xy planes. **(D–F)** The reconstruction profile along the white dotted lines in **(C)**.

The schematic of the TUS procedure is shown in Figure 1A. An ultrasound transducer (V301-SU, Olympus, United States) was attached to the mouse skull through a conical collimator filled with a bubble-free ultrasound coupling gel, which was aimed at the CA1 region of the hippocampus; the angle of the collimator to the recording electrode was approximately 45°. In our experiment, the fundamental frequency, stimulation duration, pulsed repetition frequency, and duty cycle within the stimulation, and stimulation interval were 2.25 MHz, 100 ms, 1 kHz, and 30%, and 3 s, respectively (Figure 1B). The ultrasound pressure ranged from 0.05 to 0.8 MPa. Ultrasound field distribution under the skull in the xz and xy planes were shown in Figure 1C. Reconstruction profiles were placed along the white dotted lines in the xz and xy planes (Figure 1D–F). The diameter of the focal area measured at full width at half maximum (FWHM) was ∼1.5 mm.

A computer was used to receive a multi-channel neural signal processor (Apollo, Bio-Signal Technologies, United States) through a universal serial bus connector to record the LFP signal (sampling rate: 30 kHz) from the electrodes. When the neural signal is calculated, the computer issues stimulation instructions according to the calculation results. A control signal generator (DG2052, RIGOL, China) generated the modulation signal, which was first transmitted through a linear RF power amplifier (240L, ENI Inc., United States) and then transmitted to the ultrasound transducer to emit ultrasound waves. The neural signal processor continuously collects LFP signals in real-time during the experiment, and records the ultrasound trigger signal of the signal generator at the same time. All experiments were conducted in an electromagnetic shielding cage to prevent external electromagnetic interference.

The algorithm for predicting the theta rhythm used in this experiment is based on a previous study (Kanta et al., 2019). The algorithm is programmed in MATLAB software. The computer receives the real-time LFP signals and performs downsampling (500 Hz) and filtering (4–8 Hz) with a FIR digital filter (passband frequency range: 4–8 Hz, transition stopband width: 2 Hz, stopband attenuation > 50 dB, sampling rate: 500 Hz, filter order: 826). Subsequently, the amplitude of theta is extracted using the “double threshold detection module” of the theta rhythm signal, followed by application of the Hilbert transform to extract its phase information. An ultrasound stimulation command is issued when the theta amplitude exceeds the threshold and the algorithm detects the selected phase.

### Data preprocessing

We obtained the theta (4–8 Hz) and gamma (30–45 Hz) rhythms of the LFP by filtering. A second-order IIR digital filter with a filter constant of 0.995 and a sampling frequency of 500 Hz was used for 50 Hz notch filtering on the LFP signals. The LFP signal was divided into two parts, viz. pre-stimulation (pre-stim) and post- stimulation (post-stim), for data analysis.

### Power spectrum

The LFP signal data were subjected to Welch power spectrum estimation, and the absolute power in the two frequency bands of theta (4–8 Hz) and gamma (30–45 Hz) was calculated. The total absolute power of the frequency bands (4–200 Hz) was obtained by summing the absolute powers of all frequency bands. The relative power of each frequency band was equal to the corresponding absolute power divided by the total absolute power.

### Sample entropy and Lempel-Ziv complexity

We calculated the sample entropy and Lempel-Ziv complexity of the theta and gamma rhythms, respectively. Sample entropy is a complex measure and a non-linear analysis method. The higher the sample entropy value, the greater the complexity of the signal time series. This method is especially suitable for analyzing non-stationary and non-linear LFP signals. It is calculated using the following formula:


(1) $$S\,a\,m\,p\,E\,n\,\left(m,r,N\right)=-l\,n\,\left[\frac{C^{m+1}\,\left(r\right)}{C^{m}\,\left(r\right)}\right]$$

where N is the length of the signal, m is the embedding dimension, and r is the threshold size and *m* = 2, *r* = 0.25*SD, and SD is the signal standard deviation.

Lempel-Ziv complexity is a non-linear analysis method used to characterize the degree of disorder in a time series by measuring the rate at which new patterns emerge. The formula is as follows:


(2) $$L\,Z\,C=\frac{c\,\left(n\right)\cdot l\,o\,g_{L}\,\left(n\right)}{n}$$

where n is the length of the signal, and L is the number of coarse-grained segments. In this study, *L* = 2, c(n) represents the different substrings constructed by binarizing the original LFP sequence and repeated cascading.

### Phase-amplitude coupling

PAC is used to analyze the degree of coupling between the low-frequency phase and the high-frequency amplitude. We used the phase locking value algorithm to calculate the PAC. It is calculated using the following formula:


(3) $$P\,A\,C=\left|\frac{1}{N}\,\sum_{t=1}^{N}e^{i\,\left(\varphi_{l\,o\,w}\,\left(t\right)-\varphi_{h\,i\,g\,h\,a\,m\,p}\,\left(t\right)\right)}\right|$$

where N is the length of the signal, φ_*low*_(t) is the phase of the low-frequency signal, and φ_*highamp*_(t) is the phase of the amplitude of the high-frequency signal modulated by the low frequency.

### Statistical analysis

The results were analyzed using the Kruskal–Wallis test and Mann-Whitney test. Differences were considered significant at *p*-values < 0.05. All statistical analyses were performed using the MATLAB software.

## Results

### Power spectrum of the theta and gamma rhythms evoked by peak and trough stimulation with different ultrasound pressures

**FIGURE 2:**
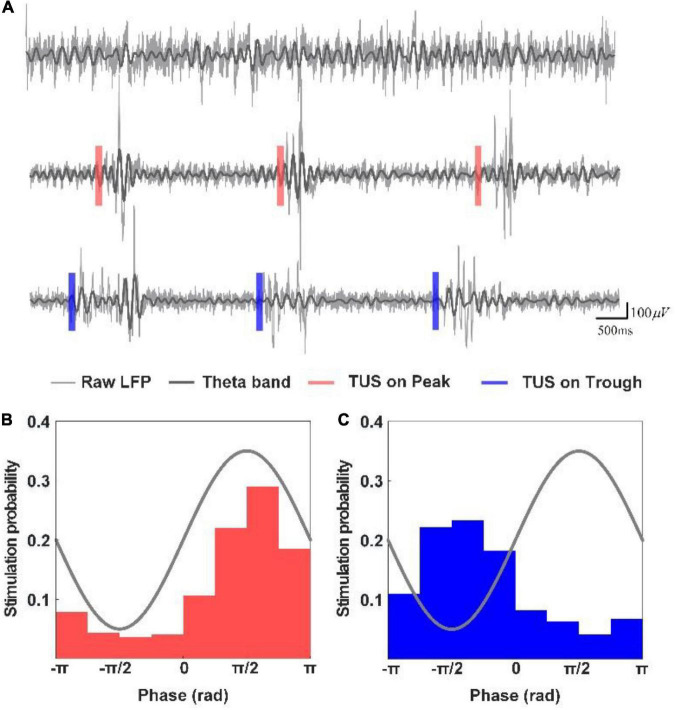
**(A)** LFPs and their corresponding theta rhythm before ultrasound stimulation, with peak stimulation and trough stimulation, respectively. **(B,C)** Phase distribution histogram of ultrasound stimulation.

First, we analyzed the changes in the power spectrum of the theta and gamma rhythms under peak and trough stimulation with different pressures. Figure 2A depicts the LFPs and their corresponding theta rhythm before ultrasound stimulation, with peak stimulation and trough stimulation, respectively. A significant increase was observed in the LFP amplitude as well as the amplitude of the theta rhythm under peak and trough stimulation, which is consistent with our previous results (Yang et al., 2020). We counted the phase of the theta rhythm corresponding to the time point when the closed-loop system sent ultrasound stimulation in the experiment, calculated the probability of occurrence of different stimulation phases, and created the phase distribution histogram of ultrasound stimulation (Figures 2B,C). We found that the phase distribution of ultrasound stimulation position in the theta rhythm is concentrated at π/2 and −π/2, respectively, which shows that the system can accurately stimulate the peak and trough of the theta rhythm.

**FIGURE 3:**
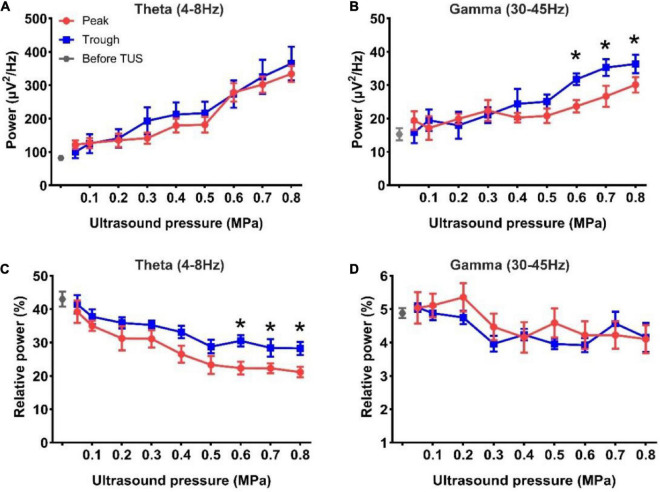
**(A,B)** The absolute power of the theta and gamma rhythms before and after TUS under peak and trough stimulations with different ultrasound pressures, **(A)** theta rhythm, **(B)** gamma rhythm. **(C,D)** The relative power of the theta and gamma rhythms before and after TUS under peak and trough stimulations with different ultrasound pressures, **(C)** theta rhythm, **(D)** gamma rhythm. (mean ± SEM, *n* = 8 for peak stimulation, *n* = 8 for trough stimulation, *p < 0.05; Kruskal–Wallis test).

We analyzed the absolute power of the theta and gamma rhythms under peak and trough stimulation. As shown in Figures 3A,B, the absolute power of the theta and gamma rhythms after TUS was higher than that before peak and trough stimulations. We observed that the absolute power of theta and gamma increased with the increase in ultrasound pressure for both peak and trough stimulations. However, the absolute power in the theta band did not differ from 0.05 to 0.8 MPa between peak and trough stimulations. There was no difference in the gamma rhythm between 0.05 and 0.5 MPa. The absolute power of the gamma rhythm under trough stimulation was higher than that under peak stimulation between 0.6 and 0.8 MPa (*p < 0.05; Kruskal–Wallis test). Figures 3C,D show the relative power of the theta and gamma rhythms. We observed that the relative power of the theta rhythm after both peak and trough stimulations was significantly lower than that before stimulation, and decreased with the increase in ultrasound pressure from 0.05 to 0.8 MPa. The relative power of the theta band under trough stimulation was significantly higher than that under peak stimulation between 0.6 and 0.8 MPa (*p < 0.05; Kruskal–Wallis test). We also noticed that the relative power of the gamma rhythm after peak and trough stimulation was mostly lower than that before stimulation, which varied with ultrasound pressure, independent of peak and trough stimulation. Last, the Pearson correlation coefficients of absolute power and relative power of LFP between peak stimulation and trough stimulation were calculated to evaluate their change trend (Absolute power, theta frequency band: 0.86 ± 0.03, gamma frequency band: 0.65 ± 0.06; Relative power, theta frequency band: 0.68 ± 0.07, gamma frequency band: 0.28 ± 0.1). The above results indicate that the absolute power and relative power of LFP have a similar trend with the increase of ultrasound pressure under peak stimulation and trough stimulation. These results demonstrate that the absolute power of the gamma rhythm and the relative power of the theta rhythm under TUS at 0.6–0.8 MPa differ between peak and trough stimulation.

### Sample entropy and complexity of the theta and gamma rhythms evoked by peak and trough stimulation with different ultrasound pressures

**FIGURE 4:**
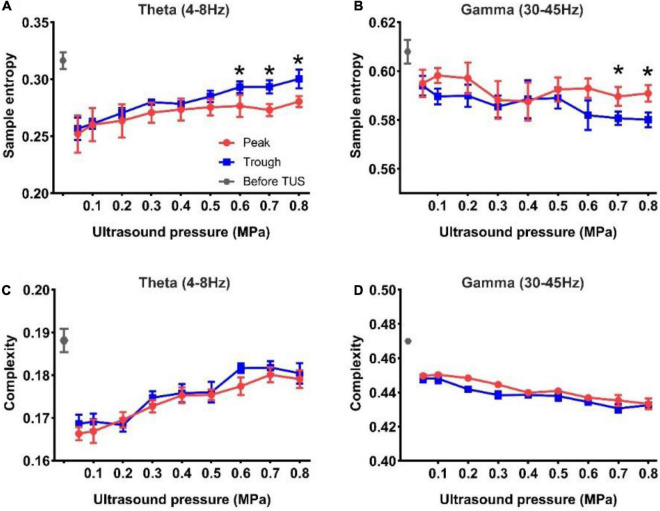
**(A,B)** The sample entropy of the theta and gamma rhythms before and after TUS under peak and trough stimulations with different ultrasound pressures, **(A)** theta rhythm, **(B)** gamma rhythm. **(C,D)** The complexity of the theta and gamma rhythms before and after TUS under peak and trough stimulations with different ultrasound pressures, **(C)** theta rhythm, **(D)** gamma rhythm. (mean ± SEM, *n* = 8 for peak stimulation, *n* = 8 for trough stimulation, *p < 0.05; Kruskal–Wallis test).

Subsequently, we analyzed the sample entropy and complexity of the theta and gamma rhythms under peak and trough stimulation with different ultrasound pressures. The results of sample entropy and complexity are shown in Figures 4A,B. We observed that the sample entropy of the theta and gamma rhythms after TUS was lower than that before TUS under both peak and trough stimulations. We also observed that the sample entropy of the theta rhythm increased, and that of the gamma rhythm decreased with the increase in ultrasound pressure under peak and trough stimulations, respectively. However, the sample entropy of the theta rhythm under trough stimulation was higher than that under peak stimulation between 0.6 and 0.8 MPa and the sample entropy of the gamma rhythm under peak stimulation was higher than that under trough stimulation from 0.7 to 0.8 MPa (*p < 0.05, ^**^p < 0.01; Kruskal–Wallis test). The complexity of the theta and gamma rhythm (Figures 4C,D) after TUS was lower than that before TUS under peak and trough stimulations. Moreover, the complexity of theta and gamma changed with ultrasound pressure, independent of peak and trough stimulation. The above-mentioned results indicate that the variation in sample entropy in the theta and gamma rhythms with ultrasound pressure is dependent on peak and trough stimulation.

### Phase-amplitude coupling between the theta and gamma rhythms evoked by peak and trough stimulation with different ultrasound pressures

**FIGURE 5:**
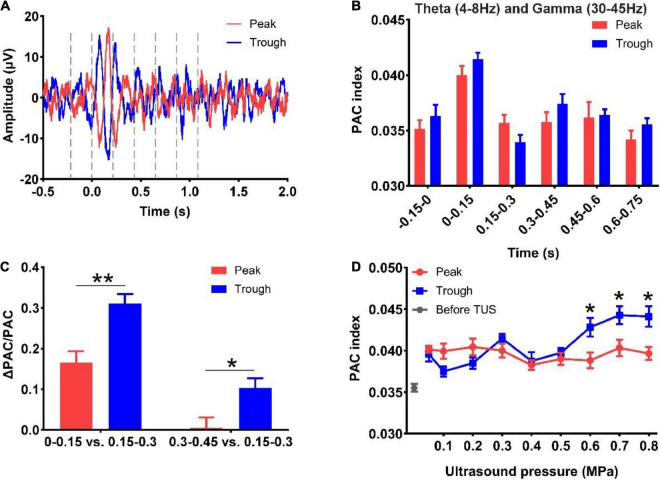
**(A)** LFP signals into six segments, viz. -0.15 to 0 s, 0–0.15 s, 0.15–0.3 s, 0.3–0.45 s, 0.45–0.6 s, and 0.6–0.75 s under peak and trough stimulation. **(B)** Phase-amplitude coupling strengths of the theta and gamma rhythms at different time points. **(C)** The relative values of phase-amplitude coupling strength at 0–0.15 s, 0.3–0.45 s, and 0.15–0.3 s under peak and trough stimulation. (mean ± SEM, *n* = 8 for peak stimulation, *n* = 8 for trough stimulation, *p < 0.05, **p < 0.01; Mann-Whitney test). **(D)** The coupling strength relative to ultrasonic pressure under peak and trough stimulations. (mean ± SEM, *n* = 8 for peak stimulation, *n* = 8 for trough stimulation, *p < 0.05; Kruskal–Wallis test).

Finally, we analyzed the PAC strength of the theta and gamma rhythms evoked by peak and trough stimulation with different ultrasound pressures. First, we divided the LFP signals into six segments, viz. −0.15 to 0 s, 0–0.15 s, 0.15–0.3 s, 0.3–0.45 s, 0.45–0.6 s, and 0.6–0.75 s under peak and trough stimulation (Figure 5A). Thereafter, the PAC strengths of the theta and gamma rhythms at different time points were calculated, as shown in Figure 5B. We found that the PAC strength at 0–0.15 s (after TUS) showed a rising trend compared to −0.15 to 0 s (before TUS) under peak and trough stimulation, albeit without statistical significance. The coupling strength increased at 0–0.15 s, decreased at 0.15–0.3 s, and increased again at 0.3–0.45 s. In order to verify whether the value of the coupling strength differed under peak and trough stimulation during these periods (0–0.15, 0.15–0.3, and 0.3–0.45 s), we calculated the relative values of coupling strength at 0–0.15, 0.3–0.45, and 0.15–0.3 s. As shown in Figure 5C, We observed that the relative values of coupling strength at 0–0.15 and 0.3–0.45 s under trough stimulation were higher than those under peak stimulation (*p < 0.05, ^**^p < 0.01; Mann-Whitney test). We analyzed the change in the coupling strength relative to ultrasonic pressure under peak and trough stimulations (Figure 5D). The coupling strength under trough stimulation was higher than that under peak stimulation between 0.6 and 0.8 MPa (*p < 0.05, ^**^p < 0.01; Kruskal–Wallis test). These results demonstrate that peak and trough stimulation affect the PAC strength between the theta and gamma rhythms as a function of ultrasound pressure.

## Discussion

In this study, we established a closed-loop ultrasound stimulation system based on the judgment of the theta rhythm peaks and troughs. Comparison of the power, sample entropy and complexity and PAC between the theta and gamma rhythms of the LFPs under peak and trough stimulation of the theta rhythm revealed the following: (1) the variation in the absolute power of the gamma rhythm and the relative power of the theta rhythm under TUS at 0.6–0.8 MPa differ between peak and trough stimulation; (2) the relationship between sample entropy of the theta and gamma rhythms and ultrasound pressure depends on peak and trough stimulation; and (3) peak and trough stimulation affect the PAC strength between the theta and gamma rhythms as a function of ultrasound pressure. To the best of our knowledge, this is the first study to demonstrate the changes in the theta and gamma rhythms with ultrasound pressure under peak and trough stimulation of the theta rhythm, which will provide the research basis for the use of ultrasound stimulation for theta- or gamma-related neural activity.

The comparison between the changes in the theta and gamma rhythms based on the stimulation pressure under closed-loop peak and trough stimulation to that under open-loop ultrasound stimulation is of considerable relevance for the following reasons. First, the relative power of the theta rhythm induced by open-loop ultrasound stimulation decreased significantly with the increase in ultrasound pressure, and there were significant differences between different pressures within 1 s after TUS (Wang et al., 2019). During closed-loop peak and trough stimulation, the relative power of the theta rhythm decreases with the increase in ultrasound pressure, which is consistent with the results of open-loop stimulation. When the ultrasound pressure was between 0.6 and 0.8 MPa, the relative power of the theta rhythm under trough stimulation was higher than that under peak stimulation. Second, in open-loop ultrasound stimulation, the relative power of the gamma rhythm increased significantly with the increase in ultrasound pressure, and there were significant differences between different pressures within 1 s of stimulation (Wang et al., 2019). During closed-loop peak and trough stimulation, the relative power of the gamma band showed a declining trend with the increase in ultrasound pressure after stimulation, but its change relative to ultrasound pressure was independent of peak and trough stimulation. Interestingly, the relative power of the gamma rhythm elicited by closed-loop peak and trough stimulation of the theta rhythm was opposite to that evoked by open-loop stimulation. Third, in open-loop ultrasound stimulation, the mean PAC strength between the theta and gamma bands increased significantly with ultrasound pressure (Yuan et al., 2016b). During closed-loop peak and trough stimulation, the PAC strength of theta and gamma did not change with the increase in the ultrasound pressure under peak stimulation, and increased with ultrasound pressure under trough stimulation. The PAC strength under trough stimulation was higher than that under peak stimulation between 0.6 and 0.8 MPa. We found marked differences in the PAC results of the theta and gamma rhythms between phase-locked closed-loop stimulation and open-loop stimulation. This comparison facilitates the increase in the number of available optional ultrasound parameters and stimulation patterns for the modulation of the theta and gamma rhythms.

In this study, we found that the changes in the theta and gamma rhythms with ultrasound pressure depended on peak and trough stimulation of the theta rhythms, but the underlying reasons were unclear. This observation may be closely related to the potential mechanism of ultrasound stimulation and the neural information contained in the peak and trough of the theta rhythm. Previous studies have shown that cholinergic neurotransmission plays an important role in the generation of theta rhythms in the hippocampus. For example, the modulation of specific receptor agonists, including the metabolic acetylcholine receptor and nicotine acetylcholine receptor agonists, can modulate theta rhythms in the hippocampus, which are determined by the activation of local neural circuits (Sun et al., 2001; Ma et al., 2020; Gao et al., 2021). In addition, the γ-aminobutyric acid type A receptor (GABAaR) also plays a key role in the modulation of the theta rhythm. For example, effective activation of GABAaR in the CA1 region of the rat hippocampus can regulate the theta rhythm (Adams et al., 2020). Gamma rhythms in the hippocampus can be induced by tonic electrical stimulation, agonists of metabotropic glutamate receptors and kainate receptors, and potassium ion solutions. Inhibitory synapses are necessary for the generation of gamma synchronization under sufficient conditions (Whittington et al., 1995; LeBeau et al., 2002; Cardin et al., 2009). Some properties of inhibitory interneuron networks are closely related to the generation of gamma oscillations. In ultrasound stimulation, ultrasound functions as a mechanical wave that can open or close mechanosensitive ion channels of neuronal cell membranes, and depolarize or hyperpolarize neurons, thereby generating neuronal action potentials (Fomenko et al., 2018; Kubanek et al., 2018; Qiu et al., 2019; Kamimura et al., 2020). Moreover, ultrasound can open TRPA1 channels in astrocytes, and Ca^2+^ influx through TRPA1 enables astrocytes to release glutamate through the Best1 channels (Oh et al., 2019). The mechanical pressure exerted by ultrasound signals (acting as mechanical waves) on neurons can significantly affect the activity of potassium-sodium mechanosensitive ion channels, including TREK-1, TREK-2, TRAAK K^+^ channels, and NaV1.5 (Sorum et al., 2021). Studies have also shown that ultrasound stimulation can promote the expression of proteins such as neurotrophic factors. In summary, since the changes in theta and gamma rhythms are affected by some receptor agonists or ion channels, we speculate that ultrasound waves affect the opening of channels through pressure, and subsequently induce changes in the theta and gamma rhythms. Furthermore, the peaks and troughs of the hippocampal theta rhythm are known to reflect differential neural information encoding, and optogenetic stimulation of the peaks and troughs produces different stimulation effects (Siegle and Wilson, 2014). Therefore, we speculate that the theta and gamma rhythms may be affected by theta rhythms stimulation with changes in ultrasound pressure. These different responses to peak and trough stimulation are closely related to the difference in neural function between the peak and trough of the theta rhythm. We endeavor to perform in-depth research to ascertain the biophysical mechanism underlying the different responses of ultrasound stimulation on peak and trough stimulation of the theta rhythm in our next study.

Several studies have reported that TUS can activate cortical neurons via auditory responses (Guo et al., 2018; Sato et al., 2018; Park et al., 2021). In a subsequent study, Mohammadjavadi et al. (2019) used ultrasound to stimulate deaf knockout mice and demonstrated that direct neural activation was caused by TUS, instead of auditory effects. Recently, Yu et al. (2021) showed that ultrasound can elicit direct responses in the rodent brain, independent of hearing. In our last research, we performed closed-loop ultrasound stimulation experiments, and found that the amplitude changes and dynamic responses on the electromyogram and LFP in normal mice were substantially similar to those in deaf mice, demonstrating that ultrasound induces motor responses and neural responses by stimulating brain tissue rather than indirect auditory effects (Yuan et al., 2022). Therefore, we speculate that the changes in theta and gamma rhythms induced by ultrasound under peak and trough stimulation are not the results of auditory effects.

Previous studies have shown that theta rhythms are related to attention to conditioned stimuli, information processing, visual search, arousal, decision-making, and memory consolidation (Kienitz et al., 2018; Fiebelkorn and Kastner, 2019; Nicolás et al., 2021). The modulation of neuronal firing and neural networks by gamma neural oscillations is closely related to the function of the nervous system. These functions of gamma neural oscillations mainly include sensation and perception, arousal, motor, attention, and memory, etc. Gamma neural oscillations play a key role in sensory feature binding, selective attention, and execution of memory tasks (Magazzini and Singh, 2018; Kanta et al., 2019; McNally et al., 2021). Our study found that the power spectrum, sample entropy and complexity, and PAC strength of the theta and gamma rhythms can be modulated by phase-locked closed-loop ultrasound stimulation. On the basis of the adjustment of these parameters, we speculate that closed-loop ultrasound stimulation based on theta rhythmicity may play a modulatory role in brain functions related to theta and gamma rhythms such as arousal, cognition, attention, memory, etc. Therefore, we can choose the ultrasound stimulation patterns that are beneficial to memory and cognition. Additionally, the theta and gamma rhythms are closely related to epilepsy, Parkinson’s disease, Alzheimer’s disease, depression, and other neurological or psychiatric diseases. This system can be used to stimulate theta rhythms of different phases to select the appropriate ultrasound stimulation pattern to improve the therapeutic effect.

## Conclusion

In conclusion, our study demonstrates that the modulation effect of ultrasound stimulation on the theta and gamma rhythms by different ultrasound pressures depends on peak and trough stimulation of the theta rhythm.

## Data availability statement

The raw data supporting the conclusions of this article will be made available by the authors, without undue reservation.

## Ethics statement

This study involving animals was reviewed and approved by the Ethics Committee of Yanshan University.

## Author contributions

YY and HJ designed and coordinated the study. ZX, JY, SD, HJ, and YY carried out the experiment and data process and drafted the manuscript. All authors gave final approval for publication.
